# Echocardiographic correlates of MRI imaging markers of cerebral small-vessel disease in patients with atrial-fibrillation-related ischemic stroke

**DOI:** 10.3389/fneur.2023.1137488

**Published:** 2023-03-23

**Authors:** Kaili Ye, Wendan Tao, Zhetao Wang, Dayan Li, Mangmang Xu, Junfeng Liu, Ming Liu

**Affiliations:** ^1^Department of Neurology, West China Hospital of Sichuan University, Chengdu, Sichuan, China; ^2^Department of Radiology, West China Hospital, Sichuan University, Chengdu, China; ^3^Cardiac Ultrasound Office, Department of Cardiology, West China Hospital, Sichuan University, Chengdu, China

**Keywords:** atrial fibrillation, echocardiographic characteristics, cerebral small-vessel disease, ischemic stroke, cardiac dysfunction

## Abstract

**Background and objectives:**

Atrial fibrillation (AF) has been linked to dementia risk, partly explained by cerebral small vessel disease (CSVD). Since AF and cardiovascular comorbidities were associated with cardiac dysfunction, we aimed to determine the association between echocardiographic parameters and neuroimaging markers of CSVD in patients with AF-related ischemic stroke.

**Methods:**

This cross-sectional study enrolled patients with AF-related ischemic stroke from March 2013 to December 2019 who underwent transthoracic echocardiography and brain 3T MRI, including T1, T2, Flair, and SWI imaging sequences. We assessed the presence of lacunes and cerebellar microbleeds (CMBs), the severity of white matter hyperintensity (WMH) scored by the Fazekas scale (0-6), and the severity of enlarged perivascular spaces (EPVS) in basal ganglia (BG) and centrum semiovale (CSO) classified into three categories (0–10, 10–25, and >25). CSVD burden was rated on a 0-to-4 ordinal scale. Generalized linear regression analysis and post hoc comparisons with Bonferroni correction were performed to assess the association between various echocardiographic parameters and these lesions, adjusted for demographics and potential confounders.

**Results:**

119 patients (68.38 ± 12.692 years; male 45.4 %) were included for analysis, of whom 55 (46.2%) had lacunes, 40 (33.6%) had CMBs, and median severity for WMH, BG-EPVS, CSO-EPVS, and CSVD burden were 2 (IQR: 1–3), 1 (IQR: 1–2), 1 (IQR: 0–1), and 1 (IQR: 1–2) respectively. In multivariable, fully adjusted models, left ventricular posterior wall thickness (LVPW) was associated with a higher risk of lacunes (RR 1.899, 95% CI: 1.342–2.686) and CSVD burden (RR = 2.081, 95%CI: 1.562–2.070). Right atrial diameter (RAD) was associated with greater CSO-EPVS (RR = 2.243, 95%CI: 1.234–4.075). No echocardiographic parameters were revealed to be associated with CMBs and WMH.

**Conclusion:**

In patients with AF-related ischemic stroke, LVPW is associated with a higher risk of lacunes and CSVD burden, while RAD was associated with greater CSO-EPVS. Larger studies are required to determine these associations and to elucidate if these associations can help facilitate cognitive evaluation and brain MRI screening.

## Introduction

Increasing evidence suggests that atrial fibrillation (AF) appears to be correlated with cognitive decline independent of clinical stroke (1, 2). Cerebral small vessel disease (CSVD) is one of the pathological mechanisms through which AF might lead to cognitive impairment. A more recent large sample of study (3) has demonstrated that nearly 25% of patients with AF-related ischemic stroke or transient ischemic attack have preexisting cognitive impairment. They found imaging markers of CSVD were independently associated with cognitive impairment prior to ischemic events.

There is no complete explanation of the mechanism that links AF and CSVD, but chronic cerebral hypoperfusion, inflammation, and shared vascular risk factors, such as hypertension and diabetes mellitus, may all be involved. Patients with AF are more likely to develop cardiac dysfunction, for example, many patients with AF develop an enlarged *left* atrium (LA) and enlarged left ventricular (LV), and are also associated with an increased incidence of heart failure. Conversely, patients with cardiac dysfunction are known to contribute to AF development and maintenance (4–6). According to previous studies, some cardiac subclinical indicators, such as left ventricular structure and LV systolic dysfunction, may contribute to greater white matter hyperintensity (WMH) (6), and LA volume, may contribute to silent brain infarcts (7) even in the absence of AF. However, none of them investigated whether cardiac structural or functional abnormalities correlated with neuroimaging markers of CSVD in patients with AF-related ischemic stroke. Thus, we evaluated the cross-sectional association of the echocardiographic parameters of cardiac structure or function with the neuroimaging markers of CSVD on MRI in patients with AF-related ischemic stroke.

## Materials and methods

### Patients and evaluation

The data that support the findings of this cross-sectional study is available from the Chengdu stroke registry, an ongoing prospective hospital-based database (8). We defined AF-related ischemic stroke when the patients had a cardioembolic stroke with AF. Acute ischaemic stroke (AIS) patients who underwent transthoracic echocardiography and the brain 3T MRI, including T1, T2, Flair, and susceptibility-weighted imaging (SWI) sequences imaging between March 2013 and December 2019 were eligible if they were 18 years or older. Patients with non-cardioembolic stroke, no evidence of AF, bilateral stroke infarcts; time from admission to stroke onset over 14 days, and unavailable MRI imaging were excluded. The diagnosis of AIS was based on the World Health Organization (9). AF was recorded as present if there was any history of it in the past, or if it was present on the electrocardiogram at the time of admission. The trial of ORG 10,172 in acute stroke treatment (TOAST) classification was used to determine AIS subtypes on the basis of their etiology (10). The echocardiogram and brain MRI were completed within 4–14 days after admission.

The study was approved by the Ethics Committee of West China Hospital, Sichuan University Scientific Research (2014[69]), and followed the Declaration of Helsinki. All patients provided written consent.

### Echocardiographic data

All patients were evaluated by 2-D transthoracic echocardiography during hospitalization, using a Philips iE33 (Philips Medical Systems, USA) echocardiography device. A single trained investigator with considerable echocardiographic experience performed all the echocardiographic evaluations blinded to other information. All measurements were performed according to the American Society of Echocardiography guidelines.

We collected echocardiographic parameters including left atrial anteroposterior diameter (LAD), left ventricular end-diastolic diameter (LVD), right anteroposterior atrial diameter (RAD), right ventricular end-diastolic diameter (RVD), interventricular septum diameter (IVS), left ventricular posterior wall diameter (LVPW), left ventricular end-diastolic diameter (LVEDD), left ventricular end-systolic diameter (LVESD), left ventricular end-diastolic volume (LVEDV), left ventricular end-systolic volume (LVESV), left ventricular cardiac and stroke volume (SV) and the left ventricular ejection fraction (LVEF) was calculated using two-plane Simpson method.

### Brain magnetic resonance imaging

MRI was performed on a 3.0-T GE scanner Discovery or Architect scanner at West China Hospital of Sichuan University. The MRI protocol included: (1) axial T1 weighted: TE = 24 ms, TR = 1,750 ms, echo train length (ETL) = 10, bandwidth (BW) = 41.67KHz, matrix = 320 × 224, slice thickness = 5 mm, filed-of-view = 240 mm, spacing = 1, number of excitations (NEX) = 1; (2) axial T2 weighted: TE = 93 ms, TR = 5,727 ms, ETL = 32, BW = 83.3 KHz, matrix = 512 × 512, filed-of-view = 240 mm, slice thickness = 5 mm, spacing = 1, NEX = 1.5; (3) FLAIR weighted: TE = 145 ms, TR = 8,400 ms, inversion time (TI) = 2,100 ms, BW = 83.3 KHz, flip angle (FA) = 145°, matrix = 320 × 224, slice thickness = 5 mm, filed-of-view = 240 mm, spacing = 1, NEX = 1; (4) Between March 2013 and May 2018, SWI weighted: TE = 20 ms, TR = 27 ms, Flip angle = 15°, slices = 64, slice thickness = 2 mm, and field of view = 185 × 220 mm); between April 2018 and December 2019, the parameters were: SWI (TE: 22 ms, TR: 38 ms, Flip angle: 15°, slices: 64, slice thickness/gap: 2/2 mm, and field of view: 240 × 216 mm).

MRI measurements were made in accordance with the consensus criteria of the Standards for Reporting Vascular changes on neuroimaging (STRIVE) (11). Lacunes were defined as deep lesions (3–15 mm) exhibiting CSF-like signals on all sequences. CMBs appeared as small, rounded, or circular hypointense lesions within the brain parenchyma with clear margins, and had a size of 2–10 mm on the SWI image. In this study, we evaluated the presence of lacunes and cerebellar microbleeds (CMBs). We evaluated lacunes and cerebellar microbleeds (CMBs) according to the presence or absence of these structures. White matter hyperplasia (WMH) was defined by FLAIR sequencing as patches of white matter in periventricular areas or the centrum semiovale area. Periventricular and deep WMHs were graded on the Fazekas scale from 0 to 3; the sum of these two parts (0 to 6) was used to calculate the total Fazekas score. Enhanced perivascular spaces (EPVSs) are defined by small punctate (if perpendicular) or linear hyperintensities (if longitudinal) on T2 images of the basal ganglia (BG) or centrum semiovale (CSO), and classified into three categories (0–10, 10–25, and >25). An ordinal score ranging from 0 to 4 was constructed to reflect the total burden of CSVD. Two trained neurologists blind to all clinical information about the study patients reviewed and analyzed the MR images.

Visualization and evaluation of MRI images were performed by a single rater (K.L.Y) blinded to clinical data. For the purpose of assessing inter-rater agreement, a second rater (W.D.T.) evaluated 25 randomly selected patients for the presence of CMBs (kappa 0.85, *p* =0.002), lacunes (kappa 0.66, *p* = 0.031), the severity of WMH (kappa 0.75, *p* = 0.002), BG-EPVS (kappa 0.62, *p* < 0.001), and CSO-EPVS (kappa 0.62, *p* < 0.001).

### Clinical measures

Information about demographics and clinical characteristics including age, gender, previous medical history (hypertension, diabetes mellitus, and hyperlipidemia), heart disease (coronary heart disease, cardiac valve disease, congestive heart failure), prior antithrombotic agents (antiplatelets, lipid-lowering, and anticoagulants) use, smoking and alcohol status. CHA2DS2-VASc score was calculated as previously described (12). Hypertension was defined by a physician's self-reported diagnosis, the use of hypertensive medications, or a measured systolic blood pressure >_140 mmHg systolic or diastolic blood pressure >_90 mmHg. Diabetes mellitus was defined by a self-reported physician's self-report, the use of hypoglycemic medications, and non-fasting serum glucose levels above 200 mg/dL, or fasting serum glucose levels above 60 mg/dL. Hyperlipidemia was defined as LDL-c >_160 mmol/dl, a history of hyperlipidemia or lipid-lowering medication. Heart disease was based on hospital discharge diagnosis codes. Coronary heart disease was defined as having a history of coronary heart disease or having been clinically diagnosed with coronary heart disease during hospitalization. Cardiac valve disease was defined as either a self-reported history of valvular disease recorded on the CRF or cardiac valve replacement, stenosis, regurgitation affecting one or more heart valves evaluated by echocardiography during hospitalization. Congestive heart failure was defined as having a history of chronic congestive heart failure.

### Statistical analysis

Data are presented as means ± SD or medians with interquartile ranges, and as proportions for categorical variables as appropriate. Inter-rater reliability of the neuroimaging variable was tested using the kappa statistic.

Univariate analysis for the association of clinical characteristics and echocardiographic parameters with MRI markers of CSVD was assessed by using generalized linear models (GLM). The Spearman χ2 test was used for categorical variables comparison. Multivariate analysis was performed to determine the contribution of echocardiographic parameters to neuroimaging burden of CSVD using generalized linear models (GLM) adjusted for age, sex, and factors with statistical significance by univariate analysis. Due to colinearity issues, both the CHA2DS2-VASc score and its sub-variables were entered into separate multivariate models. We used a regression model with a logit link function for the association of echocardiographic parameters with the presence of lacunes and CMBs. Analysis for the association of echocardiographic parameters with severity for WMH, BG-EPVS, CSO-EPVS, and CSVD burden was performed using a regression model with a probit link function. Risk ratios (RR) per standard deviation change and 95% confidence intervals (CI) were presented.

All analyses were performed using SPSS 25.0 (IBM, Armonk, New York). A *P*-value < 0.05 was considered statistically significant. Again, a Bonferroni correction (Bonferroni correction = 0.05/number of subsamples = 0.004) was applied to 12 echocardiographic variables for multiple comparisons.

## Results

### Clinical characteristics and echocardiographic parameters

**Figure 1:**
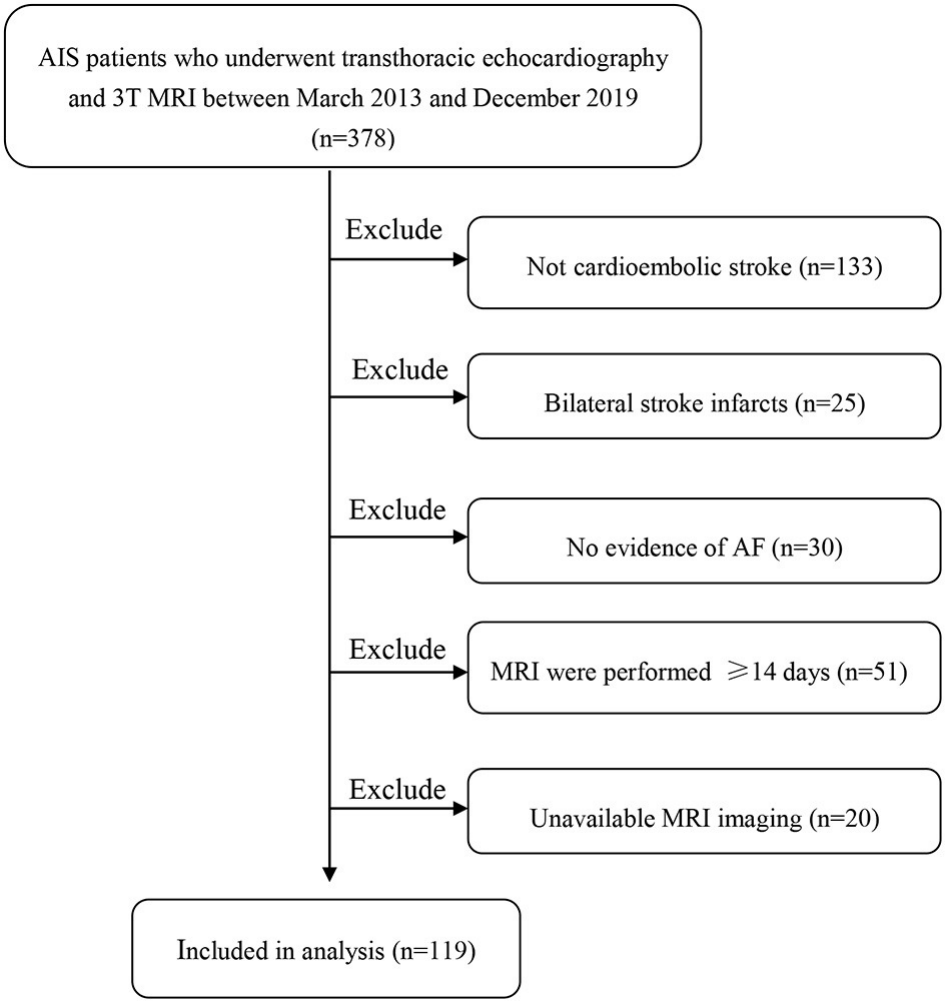
Study flowchart. AF, Atrial fibrillation; AIS, acute ischemic stroke.

A total of 378 AIS patients who underwent transthoracic echocardiography and 3T MRI were registered in the present analysis. The following patients were excluded: 133 because they had a non-cardioembolic stroke, 25 because bilateral stroke infarcts affected the identification of CSVD on neuroimaging, 30 because of no evidence of AF, and 51 because of time from admission to stroke onset over 14 days. Furthermore, 20 patients had unavailable MRI imaging, leading to the final study sample of 119 as shown in Figure 1.

The mean age of eligible patients was 68.38 ± 12.692 years, and 45.4% were male. Median CHA2DS2-VASc score was 2 (IQR: 1–3). The median time interval between echocardiogram and diagnosis of AF was 1 month (IQR: 0–36 months). The baseline characteristics and echocardiographic parameters are shown in Table 1. Among them, 55 (46.2%) had lacunes, 40 (33.6%) had CMBs, and median severity for WMH, BG-EPVS, CSO-EPVS, and CSVD burden were 2 (IQR: 1–3), 1 (IQR: 1–2), 1 (IQR: 0–1), and 1 (IQR: 1–2) respectively.

**Table 1:** Baseline characteristics and echocardiographic parameters of the study population.

**Characteristics**	**Value**
Age, years	68.38 ± 12.692
Gender, males, *n* (%)	54 (45.4)
Hypertension, *n* (%)	49 (41.2)
Diabetes, *n* (%)	14 (11.8)
Dyslipidemia, *n* (%)	4 (3.4)
Coronary heart disease, *n* (%)	7 (5.9)
Cardiac valve disease, *n* (%)	29 (24.4)
Congestive heart failure, *n* (%)	9 (7.6)
Smoking, *n* (%)	22 (18.5)
Drinking, *n* (%)	29 (24.4)
CHA2DS2-VASc score	2 (1–3)
**Previous treatments**	
Antiplatelets, *n* (%)	12 (10.1)
Lipid-lowering, *n* (%)	3 (2.5)
Anticoagulants, *n* (%)	8 (6.7)
**Echocardiographic parameters**	
LAD (mm)	41 (36–47)
LVD (mm)	46 (43–50)
RAD (mm)	42 (36–47.5)
RVD (mm)	21 (19–23)
IVS (mm)	10 (9–12)
LVPW (mm)	9 (8–11)
LVEDD (mm)	99 (85–118)
LVESD (mm)	29 (27–33)
LVEDV (ml)	99 (85–118)
LVESV (ml)	33 (27–45.5)
SV (ml)	63 (51.25–74)
LVEF (%)	66 (59–70)

Values are n (%), mean ± SD, or median (interquartile range).

CHA2DS2-VASc (congestive heart failure, hypertension, age ≥ 75 years, diabetes mellitus, stroke or transient ischemic attack (TIA), vascular disease, age 65–74 years, sex category); LAD, left atrial anteroposterior diameter; LVD, left ventricular end-diastolic diameter; RAD, right atrial anteroposterior Diameter; RVD, right ventricular end-diastolic diameter; IVS, interventricular septum diameter; LVPW, left ventricular posterior wall diameter; LVEDD, left ventricular end-diastolic diameter; LVESD, left ventricular end-systolic diameter; LVEDV, left ventricular end diastolic volume; LVESV, left ventricular end systolic volume; SV, left ventricular cardiac and stroke volume; and LVEF, left ventricular ejection fraction.

### Univariate analysis for clinical characteristics associated with MRI markers of CSVD

The univariate analysis of the clinical characteristics presented in Table 2 demonstrated that hypertension, diabetes, and CHA2DS2-VASc score were significantly associated with the risk of lacunes (all *P* < 0.05). Age, hypertension, cardiac valve disease, and CHA2DS2-VASc score were significantly associated with the risk of greater WMH (all *P* < 0.05). Age and CHA2DS2-VASc score were significantly associated with the risk of CMBs (*P* < 0.05). Besides, age, hypertension, diabetes, coronary artery disease, cardiac valve disease, CHA2DS2-VASc score, and prior anticoagulant therapy were significantly associated with BG-EPVS severity (all *P* < 0.05), while age, hypertension, cardiac valve disease, CHA2DS2-VASc score, and current drinkers were significantly associated with CSO-EPVS severity (all *P* < 0.05). Cardiac valve disease, congestive heart failure, and CHA2DS2-VASc score were significantly associated with CSVD burden (all *P* < 0.05).

**Table 2:** Univariate analysis for clinical characteristics associated with MRI markers of CSVD.

**Characteristics**	**RR (95%CI)**					
	**lacunes**	**WMH**	**CMBs**	**BG–EPVS**	**CSO–EPVS**	**CSVD burden**
Age	1.023 (0.994–1.053)	1.076 (1.045–1.107)^†^	1.040 (1.005–1.076)^†^	1.046 (1.018–1.76)^†^	1.030 (1.004–1.059)^†^	0.992 (0.97–1.018)
Gender, males	0.667 (0.322–1.382)	1.111 (0.586–2.102)	1.137 (0.530–2.439)	1.220 (0.620–2.401)	1.647 (0.837–3.245)	1.548 (0.787–3.043)
Hypertension	3.301 (1.540–7.074)^†^	2.358 (1.219–4.563)^†^	2.013 (0.929–4.361)	5.485 (2.519–11.953)^†^	2.683 (1.330–5.409)^†^	1.493 (0.757–2.945)
Diabetes	5.083 (1.339–19.300)^†^	2.280 (0.847–6.129)	1.111 (0.346–3.566)	3.117 (1.094–8.890)^†^	2.073 (0.739–5.824)	2.243 (0.818–6.153)
Dyslipidemia	1.170 (0.159–8.592)	1.080 (0.186–6.283)	0.650 (0.065–6.452)	0.281 (0.040–1.972)	0.145 (0.020–1.040)	3.487 (0.584–20.822)
Coronary heart disease	1.595 (0.341–7.457)	2.609 (0.676–10.074)	2.815 (0.598–13.243)	5.551 (1.331–22.920)^†^	3.873 (0.938–15.975)	0.782 (0.190–3.212)
Cardiac valve disease	0.430 (0.177–1.047)	0.258 (0.118–0.568)^†^	0.550 (0.212–1.425)	0.178 (0.043–0.269)^†^	0.414 (0.184–0.927)^†^	0.389 (0.163–0.930)^†^
Congestive heart failure	1.500 (0.382–5.887)	0.784 (0.243–2.622)	0.986 (0.233–4.169)	1.036 (0.290–3.691)	1.330 (0.379–4.674)	0.185 (0.038–0.896)^†^
Smoking	0.963 (0.380–2.439)	1.275 (0.563–2.889)	1.161 (0.442–3.051)	1.388 (0.584–3.297)	1.002 (0.424–2,368)	0.876 (0.368–2.088)
Drinking	1.034 (0.597–1.791)	1.181 (0.728–1.917)	1.388 (0.788–2.443)	1.247 (0.747–2.083)	2.030 (1.206–3.414)^†^	0.756 (0.450–1.269)
CHA2DS2–VASc score	1.644 (1.212–2.229)^†^	1.459 (1.252–1.699)^†^	1.463(1.079–1.985)^†^	1.499 (1.101–1.813)^†^	1.094 (1.090–1.542)^†^	0.983 (0.845–1.145)
**Previous treatments**						
Antiplatelets	0.549 (0.156–1.933)	1.603 (0.559–4.595)	0.986 (0.278–3.495)	0.966 (0.316–2.951)	0.909 (0.299–2.765)	0.916 (0.307–2.735)
Lipid–lowering	0.574 (0.051–6.508)	3.651 (0.482–27.660)	0.987 (0.087–11.226)	1.313 (0.155–11.111)	0.174 (0.018–1.626)	0.421 (0.043–3.149)
Anticoagulants	0.681 (0.155–2.988)	0.643 (0.178–2.316)	0.640 (0.123–3.326)	0.091 (0.017–0.487)^†^	0.283 (0.071–1.124)	0.286 (0.057–1.439)

Values in table are Risk Ratios (RR) per standard deviation increase and 95% confidence intervals (CI).

† p < 0.05.

CSVD, cerebral small-vessel disease; WMH, white matter hyperintensities; CMBs, cerebellar microbleeds; BG-EPVS, enlarged perivascular spaces severity in basal ganglia; and CSO-EPVS, enlarged perivascular spaces severity in centrum semiovale.

CHA2DS2-VASc (congestive heart failure, hypertension, age ≥ 75 years, diabetes mellitus, stroke or transient ischemic attack (TIA), vascular disease, age 65–74 years, sex category.

### Univariate analysis and correlation matrix of MRI markers of CSVD and echocardiographic parameters

Univariate analysis for the association of echocardiographic parameters with MRI markers of CSVD as shown in Table 3 demonstrated that LAD (RR, 0.958; 95% CI, 0.918–1.000; *P* = 0.049), IVS (RR, 1.230; 95% CI, 1.022–1.481; *P* = 0.029), and LVPW (RR, 1.738; 95% CI, 1.271–2.375; *P* = 0.001) were significantly associated with the risk of lacunes. With Bonferroni correction, the associations between LAD and IVS and lacunes were attenuated. LAD (RR, 0.958; 95% CI, 0.924–0.994; *P* = 0.021), IVS (RR, 1.195; 95% CI, 1.043–1.346; *P* = 0.012), and LVPW (RR, 1.392; 95% CI, 1.094–1.765; *P* = 0.006) were significantly associated with greater WMH, whereas the statistically significant difference in these associations was no longer significant after Bonferroni correction. LVPW (RR, 1.376; 95% CI, 1.095–1.658; *P* = 0.009) was significantly associated with BG-EPVS severity, and RAD (RR, 1.048; 95% CI, 1.011–1.087; *P* = 0.011) was significantly associated with CSO-EPVS severity, while a Bonferroni correction attenuated these associations. In addition, LAD (RR, 0.946; 95% CI, 0.907–0.985; *P* = 0.008) and LVPW (RR, 1.923; 95% CI, 1.473–2.512; *P* < 0.001) were significantly associated with CSVD burden. Bonferroni correction reduced the association between LAD and CSVD burden.

**Table 3:** Univariate analysis for association of echocardiographic parameters with MRI markers of CSVD.

**Characteristics**	**RR (95%CI)**					
	**lacunes**	**WMH**	**CMBs**	**BG–EPVS**	**CSO–EPVS**	**CSVD burden**
LAD	0.958 (0.918–1.000)	0.958 (0.924–0.994)	0.987 (0.946–1.030)	0.969 (0.926–1.014)	0.982 (0.942–1.023)	0.946 (0.907–0.985)
LVD	0.972 (0.918–1.028)	0.982 (0.935–1.030)	0.965 (0.907–1.027)	0.960 (0.900–1.022)	0.992 (0.937–1.049)	1.020 (0.969–1.071)
RAD	0.965 (0.926–1.006)	1.012 (0.990–1.054)	0.982 (0.942–1.024)	1.037 (0.991–1.073)	1.048 (1.011–1.087)	0.994 (0.961–1.027)
RVD	0.930 (0.817–1.057)	0.998 (0.901–1.100)	1.007 (0.892–1.137)	1.032 (0.918–1.150)	1.025 (0.914–1.140)	0.998 (0.894–1.097)
IVS	1.230 (1.022–1.481)	1.195 (1.043–1.346)	1.048 (0.880–1.248)	1.126 (0.953–1.300)	1.121 (0.631–1.290)	1.060 (0.909–1.216)
LVPW	1.738 (1.271–2.375)^†^	1.392 (1.094–1.765)	1.035 (0.787–1.361)	1.376 (1.095–1.658)	1.121 (0.868–1.382)	1.923 (1.473–2.512)^†^
LVEDD	0.960 (0.899–1.026)	0.963 (0.909–1.018)	0.958 (0.891–1.030)	0.938 (0.868–1.014)	0.986 (0.923–1.052)	1.029 (0.970–1.089)
LVESD	0.948 (0.885–1.015)	0.977 (0.928–1.030)	0.960 (0.893–1.003)	0.926 (0.850–1.010)	0.957 (0.891–1.028)	1.024 (0.969–1.078)
LVEDV	0.994 (0.983–1.005)	0.998 (0.989–1.007)	0.993 (0.981–1.005)	0.997 (0.985–1.008)	1.001 (0.990–1.011)	1.002 (0.993–1.011)
LVESV	0.990 (0.976–1.004)	0.998 (0.987–1.009)	0.991 (0.975–1.006)	0.994 (0.979–1.009)	0.995 (0.981–1.009)	1.001 (0.990–1.012)
SV	1.002 (0.979–1.025)	0.997 (0.976–1.017)	0.995 (0.971–1.020)	1.002 (0.977–1.027)	1.017 (0.994–1.041)	1.009 (0.987–1.030)
LVEF	1.028 (0.992–1.065)	1.003 (0.974–1.032)	1.041 (0.977–1.052)	1.020 (0.981–1.058)	1.018 (0.982–1.054)	0.974 (0.987–1.017)

Values in table are Risk Ratios (RR) per standard deviation increase and 95% confidence intervals (CI).

† p-value after Bonferroni correction < 0.004.

CSVD, cerebral small-vessel disease; WMH, white matter hyperintensities; CMBs, cerebellar microbleeds; BG-EPVS, enlarged perivascular spaces severity in basal ganglia; CSO-EPVS, enlarged perivascular spaces severity in centrum semiovale; LAD, left atrial anteroposterior diameter; LVD, left ventricular end-diastolic diameter; RAD, right atrial anteroposterior diameter; RVD, right ventricular end-diastolic diameter; IVS, interventricular septum diameter; LVPW, left ventricular posterior wall diameter; LVEDD, left ventricular end-diastolic diameter; LVESD, left ventricular end-systolic diameter; LVEDV, left ventricular end diastolic volume; LVESV, left ventricular end systolic volume; SV, left ventricular cardiac and stroke volume; and LVEF, left ventricular ejection fraction.

**Figure 2:**
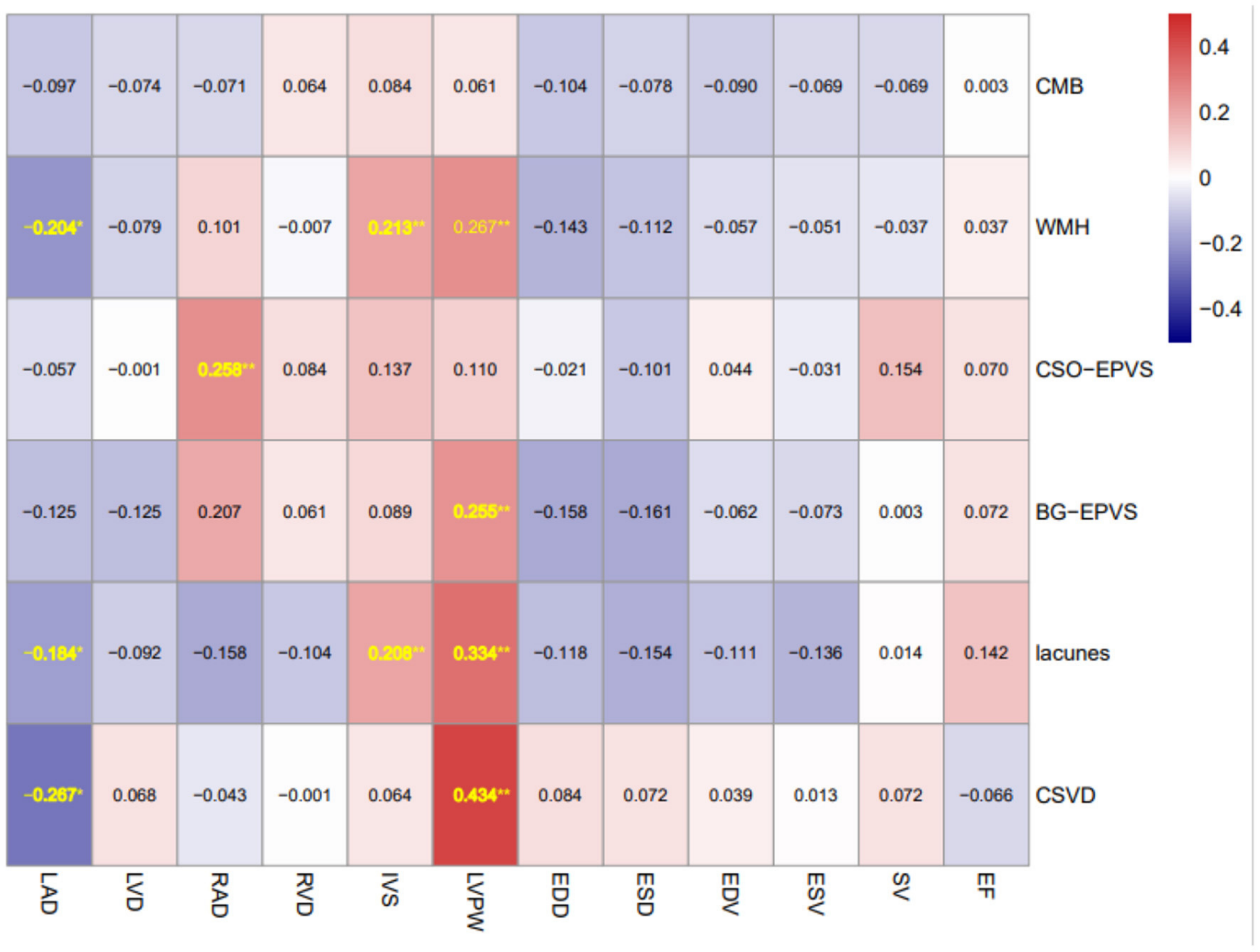
Spearman correlation matrix of MRI markers of CSVD and echocardiographic parameters. Spearman correlation matrix of MRI markers of CSVD and echocardiographic parameters (number shown in the matrix is r coefficient with statistical significance) *P < 0.05 and **P < 0.01. CSVD, cerebral small-vessel disease; WMH, white matter hyperintensities; CMBs, cerebellar microbleeds; BG-EPVS, enlarged perivascular spaces severity in basal ganglia; CSO-EPVS, enlarged perivascular spaces severity in centrum semiovale; LAD, left atrial anteroposterior diameter; LVD, left ventricular end-diastolic diameter; RAD, right atrial anteroposterior diameter; RVD, right ventricular end-diastolic diameter; IVS, interventricular septum diameter; LVPW, left ventricular posterior wall diameter; LVEDD, left ventricular end-diastolic diameter; LVESD, left ventricular end-systolic diameter; LVEDV, left ventricular end diastolic volume; LVESV, left ventricular end systolic volume; SV, left ventricular cardiac and stroke volume; and LVEF, left ventricular ejection fraction.

Spearman correlation matrix as shown in Figure 2 revealed that LAD was negatively correlated with lacunes (rs = −0.184, *P* = 0.020) and WMH (rs = −0.204, *P* = 0.047), while both IVS and LVPW were positively correlated with lacunes (rs = 0.208, *P* = 0.007; rs = 0.334, *P* < 0.001 respectively) and WMH (rs = 0.213, *P* = 0.005; rs = 0.267, *P* = 0.004 respectively). LVPW was positively correlated with BG-EPVS severity (rs = 0.255, *P* = 0.007), while RAD was positively correlated with CSO-EPVS severity (rs = 0.258, *P* = 0.002). In addition, LAD and LVPW were positively correlated with CSVD burden (rs = −0.267, *P* = 0.003; rs = 0.434, *P* < 0.001 respectively).

### Multivariate analysis for association of echocardiographic parameters with MRI markers of CSVD

Multivariate analysis included age, gender, and the variables associated with MRI markers of CSVD identified by univariate analysis and found that LVPW remained significantly associated with a higher risk of lacunes (RR = 1.899, 95%CI: 1.342–2.686, *P* < 0.001), greater WMH (RR, 1.297; 95% CI, 1.016–1.504; *P* = 0.032), and CSVD burden (RR = 2.081, 95%CI: 1.562–2.070, *P* < 0.001). RAD is significantly associated with CSO-EPVS severity (RR = 2.243, 95%CI: 1.234–4.075, *p* = 0.004). After Bonferroni correction, there was no significant difference between LVPW and greater WMH. No significant associations were observed with any echocardiographic parameters and CMBs (Table 4). These associations remained consistent after accounting for CHA_2_DS_2_-VASc Score in multivariate analysis (Supplementary Table).

**Table 4:** Multivariate analysis for association of echocardiographic parameters with MRI markers of CSVD.

**Characteristics**	**RR (95%CI)**					
	**lacunes** ^a^	**WMH** ^b^	**CMBs** ^c^	**BG–EPVS** ^d^	**CSO–EPVS** ^e^	**CSVD burden** ^f^
LAD	0.969 (0.926–1.014)	0.956 (0.913–1.001)	0.986 (0.942–1.031)	1.005 (0.955–1.057)	0.997 (0.942–1.053)	0.963 (0.918–1.010)
LVD	0.977 (0.921–1.036)	1.030 (0.977–1.085)	0.985 (0.923–1.052)	1.021 (0.963–1.083)	1.011 (0.944–1.079)	1.043 (0.987–1.098)
RAD	0.973 (0.930–1.017)	1.012 (0.998–1.047)	0.969 (0.925–1.014)	1.028 (0.991–1.067)	2.243 (1.234–4.075)^†^	1.000 (0.967–1.033)
RVD	0.941 (0.821–1.079)	1.016 (0.914–1.130)	1.012 (0.890–1.150)	1.058 (0.941–1.188)	1.030 (0.923–1.140)	0.991 (0.883–1.105)
IVS	1.152 (0.955–1.390)	1.101 (1.185–1.290)	1.008 (0.842–1.207)	1.042 (0.874–1.245)	1.021 (0.864–1,187)	1.023 (0.319–1.187)
LVPW	1.899 (1.342–2.686)^†^	1.297 (1.016–1.504)	1.012 (0.765–1.339)	1.257 (0.969–1.636)	1.149 (0.906–1.397)	2.081 (1.562–2.070)^†^
LVEDD	0.969 (0.904–1.038)	1.008 (0.949–1.070)	0.975 (0.904–1.051)	1.002 (0.938–1.071)	1.024 (0.962–1.086)	1.048 (0.985–1.112)
LVESD	0.961 (0.896–1.031)	1.013 (0.959–1.070)	0.974 (0.905–1.049)	0.995 (0.936–1.059)	0.985 (0.930–1.043)	1.040 (0.982–1.098)
LVEDV	0.999 (0.988–1.011)	1.006 (0.996–1.016)	0.996 (0.983–1.009)	1.005 (0.991–1.019)	1.003 (0.991–1.015)	1.003 (0.992–1.014)
LVESV	0.996 (0.982–1.011)	1.005 (0.994–1.017)	0.994 (0.978–1.011)	1.005 (0.988–1.021)	0.994 (0.977–1.011)	1.003 (0.990–1.016)
SV	1.000 (0.976–1.025)	1.010 (0.988–1.034)	1.002 (0.977–1.028)	1.008 (0.983–1.034)	1.025 (1.002–1.049)	1.012 (0.989–1.035)
LVEF	1.019 (0.983–1.057)	0.991 (0.962–1.021)	1.008 (0.970–1.048)	0.996 (0.963–1.030)	1.019 (0.987–1.051)	0.974 (0.943–1.008)

Values in table are Risk Ratios (RR) per standard deviation increase and 95% confidence intervals (CI).

† p-value after Bonferroni correction < 0.004.

CSVD, cerebral small-vessel disease; WMH, white matter hyperintensities; CMBs, cerebellar microbleeds; BG-EPVS, enlarged perivascular spaces severity in basal ganglia; CSO-EPVS, enlarged perivascular spaces severity in centrum semiovale; LAD, left atrial anteroposterior diameter; LVD, left ventricular end-diastolic diameter; RAD, right atrial anteroposterior diameter; RVD, right ventricular end-diastolic diameter; IVS, interventricular septum diameter; LVPW, left ventricular posterior wall diameter; LVEDD, left ventricular end-diastolic diameter; LVESD, left ventricular end-systolic diameter; LVEDV, left ventricular end diastolic volume; LVESV, left ventricular end systolic volume; SV, left ventricular cardiac and stroke volume; and LVEF, left ventricular ejection fraction.

a Adjusted for age, gender, hypertension, and diabetes mellitus.

b Adjusted for age, gender, hypertension, and cardiac valve disease.

c Adjusted for age and gender.

d Adjusted for age, gender, hypertension, diabetes mellitus, coronary heart disease, cardiac valve disease, and anticoagulants use.

e Adjusted for age, gender, hypertension, cardiac valve disease, and drinking.

f Adjusted for age, gender, cardiac valve disease, and congestive heart failure.

## Discussion

To the best of my knowledge, this is the first study carried out on the association between various echocardiographic parameters and neuroimaging burden of CSVD in patients with AF-related ischemic stroke. The main findings of our study are summarized below: (1) LVPW was associated with a higher risk of lacunes and CSVD burden. (2) RAD was associated with greater CSO-EPVS. These associations remained significant after adjusting for age, sex, and potential confounders. We emphasized the importance of echocardiographic assessment in clinical practice to facilitate cognitive evaluation and brain MRI screening of AF-related patients.

Mechanisms linking the association between LVPW and prevalent lacunes and CSVD burden in patients with AF-related ischemic stroke may involve various aspects, as follows. First, this association may be partially mediated by abnormal perfusion of the brain microvasculature. Compared to sinus rhythm, AF could trigger a higher variability, which would lead to critical cerebral hemodynamic events (hypoperfusion or hypertensive events) at the capillary level during AF (13). Cardiac changes of LVPW can contribute to AF development and maintenance, which could in turn disrupt the perfusion of the brain microvasculature. Another potential explanation linking an increase in LVPW and cerebrovascular disease could be at least explained by shared risk factors, which is in line with Michelle et al. (14). Previous study indicated that greater LVPW is common in longstanding hypertension (15), and thus could be considered a potent marker of cardiovascular risk. The greater LVPW may be not directly associated with lacunes and CSVD burden but rather indirectly as an index of exposure to cardiovascular risk factors. In addition, these brain injuries may also be caused by microemboli resulting from this cardiac abnormality. Further study with sufficient data to speculate about the likelihood of these alternative mechanisms is needed.

Another interesting finding of the present study was that RAD was associated with greater CSO-EPVS. Fewer studies involved the association between right atrium diameter and neuroimaging markers of CSVD previously. In fact, AF can also result in enlargement and fibrosis of the right atrium, which may interfere with the function of the right atrium (16, 17). Right atrium enlargement is usually accompanied by elevated right atrial pressure, according to research (18). Under pathological conditions, the enlarged right atrium may contribute to an elevation in the volume and pressure of systemic circulation veins, and even result in cerebrovenous congestion and obstruction of glymphatic drainage from the perivascular spaces (19). Perivascular spaces are potential spaces filled with interstitial fluid that flow along cerebral arteries and veins, which have been shown to play an important role in the clearance of interstitial fluid and waste products from the brain into the meningeal and cervical lymphatics (20). Insufficient glymphatic clearance caused by an enlarged right atrium may ultimately lead to toxic metabolic accumulation and neurovascular inflammation (21). According to one study (22) of chronic valvular heart disease patients, right atrial pressure is independently associated with higher WMH volume. RAD has also been reported (23) to be negatively correlated with mean deep regional CBF. Collectively, there is therefore a need to continuously pay attention to pathogenesis involving RAD in CSVD in future research.

Our study should be considered in light of several limitations. Firstly, due to the current study's cross-sectional design, we cannot examine causal relationships between echocardiographic characteristics and neuroimaging markers of CSVD in AF-related patients, but only document associations. Secondly, echocardiography is known to have limited precision, while the cost and ease of echocardiography make it an ideal examination for admission to a hospital by individuals. Thirdly, patients with permanent or persistent AF may have very different echocardiographic parameters to those with paroxysmal and that has been shown in several prior papers, while the retrospective design represents a limitation of the current study to obtain information on the type of AF. Lastly, it was done in a single center with a relatively small number of patients which may cause selection bias.

## Conclusion

In patients with AF-related ischemic stroke, LVPW is associated with a higher risk of lacunes and CSVD burden, while RAD was associated with greater CSO-EPVS. Further studies with a larger sample size and a longitudinal design were needed to continue to determine these associations and to elucidate if these associations can help facilitate cognitive evaluation and brain MRI screening.

## Data availability statement

The original contributions presented in the study are included in the article/Supplementary material, further inquiries can be directed to the corresponding author.

## Ethics statement

The study was approved by the Ethics Committee of West China Hospital, Sichuan University Scientific Research. The patients/participants provided their written informed consent to participate in this study.

## Author contributions

KY: study concept, data analysis, statistics, and paper writing. WT: study concept, patient recruitment, data analysis, statistics, and study protocol development. ZW and DL: imaging data acquisition and interpretation of data. MX: study protocol design and data analysis. JL: data analysis and informed consent acquisition. ML: study concept and guidance. All authors contributed to the article and approved the submitted version.
