# Supplementary material for: Echocardiographic correlates of MRI imaging markers of cerebral small-vessel disease in patients with atrial-fibrillation-related ischemic stroke
Source: Front Neurol. 2023 Mar 23;14:1137488. doi: 10.3389/fneur.2023.1137488 (PMC10076729; doi:10.3389/fneur.2023.1137488)
Supplement: Supplementary file 1 [file Table_1.docx]

**SUPPLEMENTARY TABLE. Multivariate analysis for association of echocardiographic parameters with MRI markers of CSVD**

| **Characteristics** | **RR (95%CI)** | | | | | |
| --- | --- | --- | --- | --- | --- | --- |
|  | **lacunes^a^** | **WMH^b^** | **CMBs^c^** | **BG-EPVS^d^** | **CSO-EPVS^e^** | **CSVD burden^f^** |
| **LAD** | **0.960 (0.917-1.004)** | **0.984 (0.954-1.014)** | **0.991 (0.948-1.037)** | **1.006 (0.966-1..046)** | **1.012 (0.966-1.058)** | **0.977 (0.949-1.006)** |
| **LVD** | **0.998 (0.939-1.060)** | **1.016 (0.978-1.055)** | **0.985 (0.923-1.051)** | **1.014 (0.957-1.070)** | **1.016 (0.961-1.071)** | **1.024 (0.984-1.064)** |
| **RAD** | **0.957 (0.914-1.003)** | **1.007 (0.984-1.029)** | **0.979 (0.936-1.023)** | **1.016 (0.986-1.045)** | **1.050 (1.015-1.085)†** | **0.997 (0.973-1.020)** |
| **RVD** | **0.953 (0.833-1.089)** | **1.040 (0.969-1.111)** | **1.034 (0.912-1.172)** | **1.032 (0.927-1.136)** | **1.044 (0.934-1.154)** | **1.002 (0.926-1.081)** |
| **IVS** | **1.172 (0.969-1.417)** | **1.112 (1.010-1.215)** | **1.001 (0.834-1.203)** | **1.023 (0.905-1.146)** | **1.039 (0.882-1.225)** | **1.011 (0.910-1.122)** |
| **LVPW** | **1.758 (1.278-2.417)†** | **1.182 (1.026-1.337)** | **1.017 (0.768-1.347)** | **1.256 (1.043-1.514)** | **1.175 (0.919-1.502)** | **1.439 (1.236-1.673)†** |
| **LVEDD** | **0.993 (0.925-1.066)** | **1.008 (0.949-1.070)** | **0.977 (0.905-1.055)** | **1.014 (0.957-1.072)** | **1.031 (0.968-1.095)** | **1.023 (0.983-1.064)** |
| **LVESD** | **0.971 (0.906-1.041)** | **1.014 (0.975-1.053)** | **0.976 (0.906-1.051)** | **0.996 (0.943-1.049)** | **0.985 (0.930-1.044)** | **1.015 (0.979-1.051)** |
| **LVEDV** | **0.998 (0.987-1.009)** | **1.006 (0.996-1.016)** | **0.996 (0.984-1.008)** | **1.001 (0.991-1.021)** | **1.002 (0.992-1.012)** | **1.003 (0.992-1.014)** |
| **LVESV** | **0.994 (0.980-1.008)** | **1.003 (0.996-1.009)** | **0.994 (0.978-1.009)** | **0.997 (0.982-1.012)** | **0.995 (0.982-1.007)** | **1.001 (0.993-1.010)** |
| **SV** | **1.012 (0.986-1.037)** | **1.007 (0.992-1.021)** | **1.001 (0.976-1.028)** | **1.013 (0.992-1.034)** | **1.028 (1.005-1.051)** | **1.009 (0.994-1.024)** |
| **LVEF** | **1.022 (0.985-1.061)** | **1.001 (0.977-1.024)** | **1.008 (0.971-1.047)** | **1.009 (0.976-1.043)** | **1.021 (0.989-1.053)** | **0.993 (0.969-1.017)** |

**Values in table are Risk Ratios (RR) per standard deviation increase and 95% confidence intervals (CI). † p value after Bonferroni correction <0.004**

**Abbreviation: CSVD, cerebral small-vessel disease; WMH, white matter hyperintensities; CMBs, cerebellar microbleeds; BG-EPVS, enlarged**

**perivascular spaces severity in basal ganglia; CSO-EPVS, enlarged perivascular spaces severity in centrum semiovale; LAD, left atrial**

**anteroposterior diameter; LVD, left ventricular end-diastolic diameter; RAD, right atrial anteroposterior diameter; RVD, right ventricular**

**end-diastolic diameter; IVS, interventricular septum diameter; LVPW, left ventricular posterior wall diameter; LVEDD, left ventricular**

**end-diastolic diameter; LVESD, left ventricular end-systolic diameter; LVEDV, left ventricular end diastolic volume; LVESV, left ventricular**

**end systolic volume; SV, left ventricular cardiac and stroke volume; and LVEF, left ventricular ejection fraction.**

**^a^Adjusted for CHA2DS2-VASc score**

**^b^Adjusted for CHA2DS2-VASc score and cardiac valve disease**

**^c^Adjusted for CHA2DS2-VASc score**

**^d^Adjusted for CHA2DS2-VASc score, coronary heart disease, cardiac valve disease, and anticoagulants use**

**^e^Adjusted for CHA2DS2-VASc score, cardiac valve disease, and drinking**

**^f^Adjusted for CHA2DS2-VASc score and cardiac valve disease**
